# Selection of optimal molecular targets for tumor-specific imaging in pancreatic ductal adenocarcinoma

**DOI:** 10.18632/oncotarget.18232

**Published:** 2017-05-26

**Authors:** Willemieke S. Tummers, Arantza Farina-Sarasqueta, Martin C. Boonstra, Hendrica A. Prevoo, Cornelis F. Sier, Jan S. Mieog, Johannes Morreau, Casper H. van Eijck, Peter J. Kuppen, Cornelis J. van de Velde, Bert A. Bonsing, Alexander L. Vahrmeijer, Rutger-Jan Swijnenburg

**Affiliations:** ^1^ Department of Surgery, Leiden University Medical Center, Leiden, The Netherlands; ^2^ Department of Pathology, Leiden University Medical Center, Leiden, The Netherlands; ^3^ Department of Surgery, Erasmus Medical Center, Rotterdam, The Netherlands

**Keywords:** molecular imaging, pancreatic cancer, biomarker

## Abstract

Discrimination of pancreatic ductal adenocarcinoma (PDAC) from chronic pancreatitis (CP) or peritumoral inflammation is challenging, both at preoperative imaging and during surgery, but it is crucial for proper therapy selection. Tumor-specific molecular imaging aims to enhance this discrimination and to help select and stratify patients for resection. We evaluated various biomarkers for the specific identification of PDAC and associated lymph node metastases. Using immunohistochemistry (IHC), expression levels and patterns were investigated of integrin αvβ6, carcinoembryonic antigen-related cell adhesion molecule 5 (CEACAM5), Cathepsin E (Cath E), epidermal growth factor receptor (EGFR), hepatocyte growth factor receptor (c-MET), thymocyte differentiation antigen 1 (Thy1), and urokinase-type plasminogen activator receptor (uPAR). In a first cohort, multiple types of pancreatic tissue were evaluated (n=62); normal pancreatic tissue (n=8), CP (n=7), PDAC (n=9), tumor associated lymph nodes (n=32), and PDAC after neoadjuvant radiochemotherapy (n=6). In a second cohort, tissues were investigated (n=55) with IHC and immunofluorescence (IF) for concordance of biomarker expression in all tissue types, obtained from an individual patient. Integrin αvβ6 and CEACAM5 showed significantly higher expression levels in PDAC versus normal pancreatic tissue (P=0.001 and P<0.001, respectively) and CP (P=0.003 and P<0.001, respectively). Avβ6 and CEACAM5 expression identified tumor-positive lymph nodes correctly in 84% and 68%, respectively, and in 100% of tumor-negative nodes for both biomarkers. In conclusion, αvβ6 and CEACAM5 are excellent biomarkers to differentiate PDAC from surrounding tissue and to identify lymph node metastases. Individually or combined, these biomarkers are promising targets for tumor-specific molecular imaging of PDAC.

## INTRODUCTION

Pancreatic ductal adenocarcinoma (PDAC) is predicted to become the second cause of cancer-related death in 2030 [1]. Patients with PDAC have a dismal prognosis, with a five-year survival rate of less than 5% [2]. At this point, complete surgical resection is the only potential curative treatment. However, at the time of primary diagnosis, 75% to 85% of patients has advanced unresectable disease due to locoregional spread and metastasis [3, 4]. Therefore, accurate identification of potential candidates for resection is crucial to prevent unnecessary surgical risks and delay in systemic therapy [5]. Another challenge during surgery, is that despite careful selection of resectable patients using CT, MRI and/or PET imaging, incomplete (R1) resection occurs in up to 70% of cases [6]. Failure to identify tumor-positive margins during surgery is not surprising, and is due to the tumor's characteristic to quickly spread beyond the pancreas via perineural and perivascular pathways [7] and the inability of the surgeon to differentiate between tumor and (peritumoral) inflammatory pancreatic tissue [8, 9]. This is also challenging in pre-operative imaging with conventional technique since both PDAC and chronic pancreatitis (CP) may present similar due to abundant stroma.

To improve surgical outcomes, novel neoadjuvant treatment protocols, such as FOLFIRINOX, are being successfully implied in the treatment of PDAC. Unfortunately, a drawback of these neoadjuvant therapies is that current imaging modalities are unable to differentiate between vital tumor and radiochemotherapy-induced tumor necrosis and fibrosis [10–12]. In addition, neoadjuvant treatment effects worsen the ability of the surgeon to differentiate (vital) tumor from fibrotic pancreatic tissue and often times, serial frozen section analysis is required to assess whether to continue a resection.

Tumor imaging using molecularly targeted probes has the potential to play an important role in improving patient management and treatment in these situations. This technique can be used for tumor detection, characterization, staging, and response assessment to neoadjuvant treatment. Moreover, it can facilitate image-guided therapy, and provide surgical guidance during tumor resection [13]. Recently, a first-in-human study was conducted using intra-operative tumor-specific imaging. A targeting ligand combined with a fluorophore was used for the detection of tumor-specific biomarkers for diagnostic imaging and surgical decision-making in ovarium cancer [14].

For tumor-specific imaging, a biomarker is required to function as a target. In PDAC, a large number of biomarkers are known to be overexpressed. However, a limited number of these markers are eligible candidates for targeted imaging. Potential biomarkers for tumor-specific targeting must possess certain characteristics, such as homogeneous expression, upregulation of more than ten times compared to normal and surrounding tissue, and localization on the cellular membrane [15–17]. A preliminary study from our own research group identified several potential targets which are overexpressed in pancreatic tumor compared to normal pancreatic tissues; integrin αvβ6, carcinoembryonic antigen (CEA), epidermal growth factor receptor (EGFR), and urokinase plasminogen activator receptor (uPAR) [18]. These biomarkers have also shown their potential for tumor-targeted imaging in pre-clinical studies [15, 19–31]. However, a potential target for clinically relevant differentiation between PDAC and peritumoral inflammation or CP has yet to be identified. Next to the ability to discriminate PDAC from CP, the target also needs to be able to identify lymph node metastases and differentiate between vital tumor cells versus necrosis and fibrosis in patients that have received neoadjuvant therapy.

The primary aim of this study was to evaluate the ability of integrin αvβ6, carcinoembryonic antigen-related cell adhesion molecule 5 (CEACAM5), Cathepsin E (Cath E), EGFR, hepatocyte growth factor receptor (c-MET), thymocyte differentiation antigen 1 (Thy1), and uPAR as targets to differentiate between PDAC, CP and normal pancreatic tissue for tumor-specific imaging and for the potential to develop clinically translatable imaging agents targeting these markers. The biomarkers were selected based on a literature search and our previous study using tissue microarrays (TMA) of 137 patients [18, 19, 32, 33].

## RESULTS

In the first cohort, a total of 62 tissues (n=8 PDAC, n=7 CP, n=9 normal pancreatic tissue, n=32 lymph nodes, n=6 PDAC after neoadjuvant radiochemotherapy) were stained for all biomarkers to assess expression patterns. In the second cohort, a total of 55 tissue slides were stained with the two most suitable biomarkers (n=12 PDAC, n=12 CP, n=12 normal pancreatic tissue, n=19 lymph nodes). Patient and tumor characteristics are summarized in Supplementary Table 1.

### Avβ6, CEACAM5, CathE and uPAR are significantly higher expressed in PDAC versus CP

**Figure 1:**
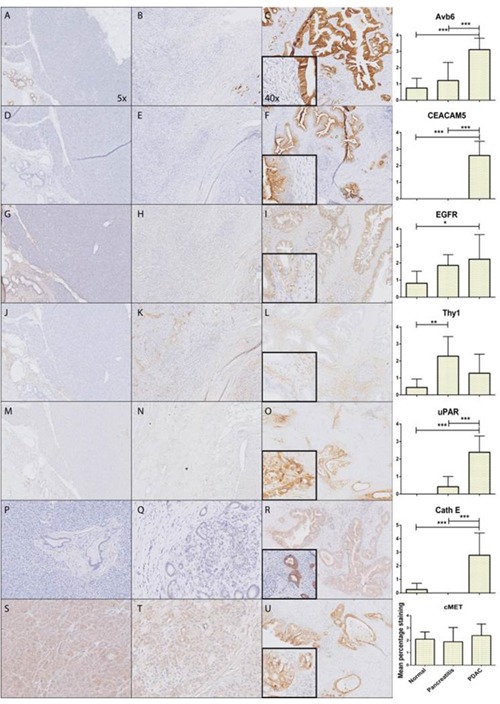
Expression patterns of investigated markers Representative images of immunohistochemically staining patterns in PDAC of all molecular markers; αvβ6 **(A-C)**, CEACAM5 **(D-F)**, EGFR **(G-I)**, Thy1 **(J-L)**, uPAR **(M-O)**, CathE **(P-R)**, cMET **(S-U)** showing respectively from left to right normal pancreatic tissue, CP, PDAC and graphical representation of mean percentage staining on all the tissue slides (*: p<0.05, **: p<0.01, ***: p<0.001).

All biomarkers were overexpressed in PDAC, except for c-MET (Figure 1S-1U) and Thy 1 (Figure 1J-1L), which showed equal or even higher staining in normal pancreatic tissue or CP compared to PDAC. Although there was abundant Thy1 stromal staining in PDAC, it was also present in stroma of adjacent normal and inflamed pancreatic tissue. Therefore, both targets were excluded from further analysis. All other targets showed significant higher expression in PDAC compared to normal pancreatic tissue; αvβ6 (Figure 1A-1C) (p <0.001), CEACAM5 (Figure 1D-1F) (p <0.001), EGFR (Figure 1G-1I) (p <0.05), Cath E (Figure 1P-1R) (p <0.001), and uPAR (Figure 1M-1O) (p <0.001). Avβ6 was homogeneously expressed, uPAR, and Cath E were slightly heterogeneously expressed, and all other biomarkers showed a markedly heterogeneous expression pattern in PDAC tissue (Supplementary Figure 1). Furthermore, only CEACAM5 expression in PDAC was slightly more intense in the invasive border compared to the tumor core. The following biomarkers were significantly overexpressed in PDAC versus CP; αvβ6 (Figure 1B-1C) (p<0.001), CEACAM5 (Figure 1E-1F) (p <0.001), Cath E (Figure 1Q-1R) (p <0.001), and uPAR (Figure 1N-1O) (p <0.001). All targets, except for CEACAM5, showed expression in the duodenum especially at the luminal side (not shown). This was also the case in colonic tissue where there was luminal staining for most targets except for uPAR (not shown).

Interestingly, immunohistochemical staining for CEACAM5 (Supplementary Figure 2) and uPAR showed complete absence of staining in normal pancreatic parenchyma. Avβ6 showed moderate staining in normal ductal structures, but there was a significantly higher expression in malignant ducti. The substantially higher ratio of ductal structures in malignant pancreatic tissue compared to normal pancreatic parenchyma adds to the significant increase of marker expression per high power field. (Supplementary Figure 3).

### Avβ6 specifically differentiates tumor-positive and tumor-negative lymph nodes

**Figure 2:**
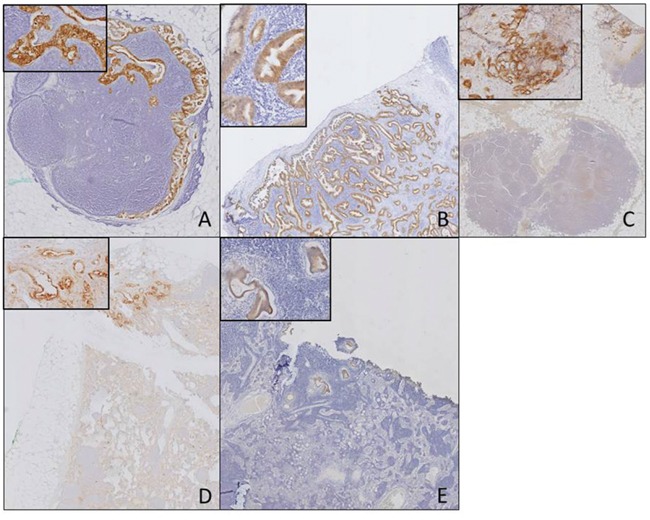
Biomarker expression in tumor-positive lymph nodes Biomarker expression in tumor-positive lymph nodes for the different biomarkers αvβ6 **(A)**, CEACAM5 **(B)**, EGFR **(C)**, uPAR **(D)**, CathE **(E)** (Objective 1x, insert 20x).

Next, we investigated biomarker expression in tumor-positive and –negative lymph node. A total of 17 tumor-positive and 15 tumor-negative nodes were stained. The described biomarkers identified the investigated tumor-positive and -negative lymph nodes correctly with, respectively, a sensitivity and specificity of; αvβ6 (84%; 100%), CEACAM5 (68%; 100%), EGFR (93%; 67%), Cath E (54%; 83%), and uPAR (69%; 67%). Figure 2 shows biomarker expression in tumor-positive lymph nodes for all investigated biomarkers.

An interim analysis to assess the suitability of the biomarker panel as targets for tumor-specific molecular imaging of PDAC was performed, shown in Table 1. Biomarkers αvβ6 and CEACAM5 met most of the criteria of an optimal tumor-specific biomarker and were selected for further evaluation.

**Table 1:** Suitability of biomarkers

Target	Localization	Upregulation in PDAC compared to normal	Upregulation in PDAC compared to CP	Sensitivity of target for lymph node metastases	Diffuse upregulation throughout the tumor	Main advantage	Main disadvantage
α_v_β_6_	Cell membrane	p<0.001	p<0.001	84%	++	Diffuse, strong expression.	Expression on normal ductal structures.
CEACAM5	Cell membrane	p<0.001	p<0.001	68%	+	No expression in normal pancreatic tissue and CP.	Heterogeneous expression and loss of expression after neoadjuvant treatment.
EGFR	Cell membrane	p=0.031	p=1.000	93%	++	EGFR-mediated cellular internalization of targeted particles.	Relatively high expression on normal duodenal tissue, thereby changing surgical field.
uPAR	Cell membrane	p<0.001	p<0.001	69%	++	Expression both on tumor and tumor surrounding stromal cells.	High uPAR expression in negative lymph nodes.
CathE	Intracellular	p<0.001	p<0.001	54%	+	Ideal for imaging purposes due to possible use of activatable probes, and therefore increasing signal-to-background ratio.	Intracellular location of target requires a probe capable of internalization.

Overview of the characteristics of the analyzed biomarkers for determination of the suitability of the biomarker as tumor-target for molecular-imaging during pancreatic cancer surgery; Integrin A_v_β_6_, carcinoembryonic antigen (CEACAM5), epidermal growth factor receptor (EGFR), hepatocyte growth factor receptor (or c-MET), urokinase-type plasminogen activator receptor (uPAR), and Cathepsin E (Cath E). No upregulation (-). Heterogeneous upregulation in tumor (+). Diffuse expression of target (++).

### Biomarker patterns change after neoadjuvant radiochemotherapy

**Figure 3:**
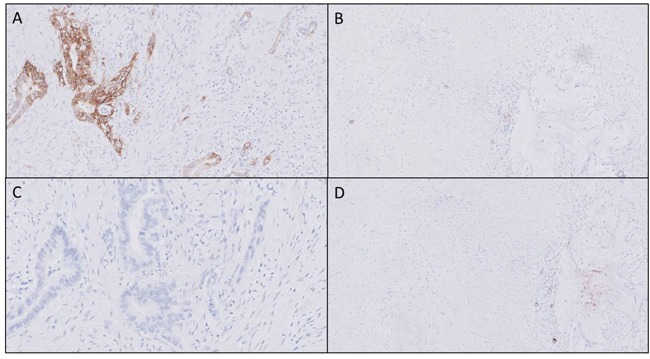
Biomarker expression after neoadjuvant therapy In all neoadjuvant treated tissue a markedly higher αvβ6 expression was identified in vital tumor tissue **(A)** compared to surrounding fibrosis or necrosis **(B)** after neoadjuvant therapy. CEACAM5 biomarker expression showed increased heterogeneity after neoadjuvant therapy in the tumor **(C)**, therefore clear distinction between vital tumor and fibrosis and necrosis is not possible **(C and D)** (Objective 10x).”

Molecular targeted imaging could be useful tool for neoadjuvant therapy response monitoring. The consequence of neoadjuvant therapy on αvβ6 and CEACAM5 expression was assessed by staining tissues from patients who received radiochemotherapy. In all investigated neoadjuvantly treated tumors, there was a markedly higher αvβ6 expression seen in the remaining vital tumor cells compared to the surrounding fibrosis and necrosis, as shown in Figure 3A and 3B. This level of expression was comparable to the expression in PDAC without neoadjuvant therapy, as previously shown. CEACAM5 expression in vital tumor cells after neoadjuvant therapy was reduced, as shown in Figure 3C, compared to PDAC not treated neoadjuvantly. As a result, no difference in expression of CEACAM5 between vital tumor cells and surrounding fibrosis and necrosis was seen (Figure 3C and 3D). In the samples of two out of six patients there was no expression of CEACAM5 in the remaining vital PDAC cells. Due to small sample size, we were not able to determine significance of results.

### Avβ6 and CEACAM5 expression is markedly different between normal pancreatic parenchyma, peritumoral inflammation, and PDAC of individual patients

**Figure 4:**
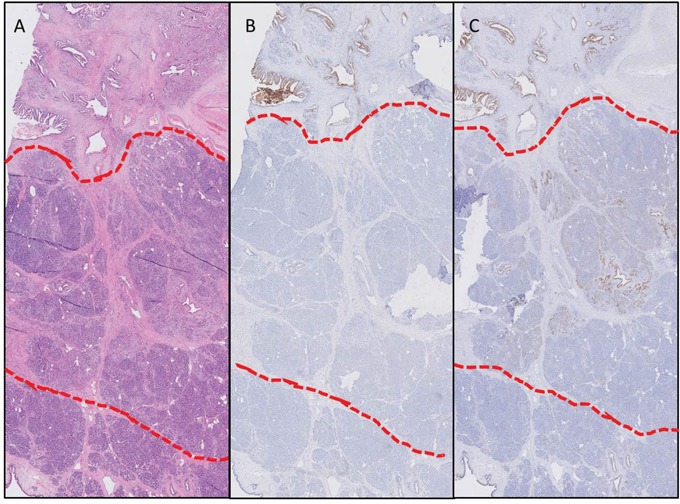
Biomarker expression in different tissue types within one patient Expression of CEACAM5 **(B)** and αvβ6 **(C)** of one individual patient with the corresponding H&E slide **(A)** in PDAC (above red line), peritumoral inflammation (between red lines), and normal pancreatic parenchyma (below red lines), showing the difference in expression even in such a close proximity (Objective 2x).”

In a second cohort, normal pancreatic parenchyma, peritumoral inflammation and PDAC of individual patients (n=12) were included in order to mimic the actual clinical situation. In 11 out of 12 patients, αvβ6 staining in PDAC was clearly higher compared to inflammation and to normal pancreatic parenchyma (4 and 2 times higher, respectively). CEACAM5 expression was only present in PDAC, and therefore, the staining pattern was markedly different between normal pancreatic parenchyma, peritumoral inflammation and PDAC in all patients except for two who did not express CEACAM5 in any of their tissue samples (Supplementary Figure 4). In concordance with the first patient cohort, CEACAM5 staining of PDAC was weaker and more heterogeneous compared to αvβ6. In Figure 4, expression of CEACAM5 (Figure 4B) and αvβ6 (Figure 4C) in PDAC, peritumoral inflammation, and normal pancreatic parenchym of one individual patient are presented next to the corresponding H&E stain (A), showing the difference in expression even in such close proximity.

### Multiplexing for optimal discrimination between malignant and benign tissue

**Figure 5:**
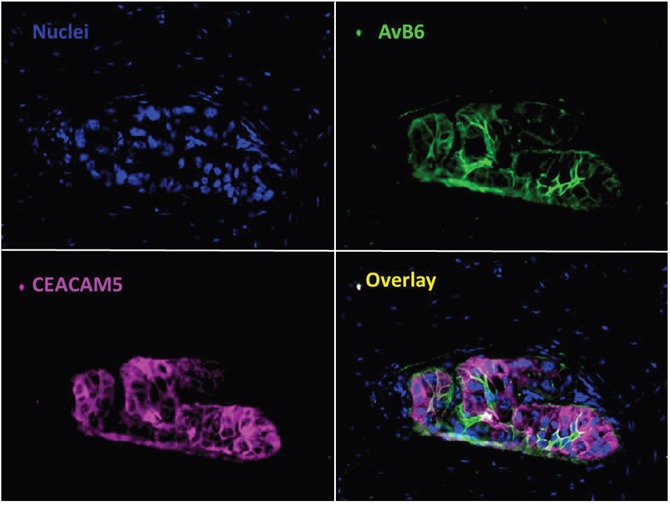
Value of multiplexing in PDAC Immunofluorescent staining of αvβ6 and CEACAM5 simultaneously in PDAC. Showing the additional value of using two biomarkers to target all tumor tissue compared to one biomarker (Objective 40x).

Double staining of CEACAM5 and αvβ6 using immunofluorescence showed that multiplexing by targeting both biomarkers leads to an improved discrimination between malignant and benign pancreatic tissue and identification of all PDAC tissue. Examples of using multiplexing to achieve improved detection are shown in Figure 5, and Supplementary Figure 5. This improved detection is obtained by the different expression patterns of both targets, as also shown in Figure 1, which are complementary to each other. Targeting αvβ6 will identify all PDAC as shown above, and CEACAM5 reduce the false positive staining by αvβ6 in normal pancreatic parenchyma and CP.

## DISCUSSION

Imaging using tumor-specific molecular probes has the potential to solve some of the challenges regarding PDAC diagnosis, treatment response monitoring and surgery. For tumor-specific molecular imaging to be successful, it is essential to have suitable biomarkers that can be targeted by molecular probes and are able to distinguish primary PDAC and metastases from normal pancreatic tissue, CP or acute (peritumoral) inflammation. With the current imaging modalities, the differentiation between PDAC and CP remains a challenge as still 7-13% of pancreatectomies are performed for benign pathology [34]. Molecular imaging can potentially also improve the outcome of PDAC patients by providing the ability to identify small tumor nodules in an early stage and therefore increasing the chance of cure and improving survival rates [35, 36]. Another major problem for PDAC surgery is the high recurrence rate within 6-12 months, which suggests that local or distant micrometastases were already present at the time of surgery [37, 38]. This underscores that conventional imaging modalities (i.e., MRI, CT and PET) lack the sensitivity to detect small amounts of tumor leading to highly morbid surgical procedures for patients without the desired oncologic benefit. A technique that improves the stratification of patients to ensure receivement of the proper therapy could serve as a helpful tool for personalized treatment in PDAC. Examples of tumor-specific imaging modalities include: ultrasound, CT, PET, and fluorescent optical imaging. Fluorescent optical imaging has shown the ability to detect lesions <2mm as described by Warram, et al. [39]. Of course, this modality can only be used during surgery but will lead to better stratification intraoperatively.

For PDAC, the greatest improvements in patient outcome would likely be the result of better selection of patients for surgery, preoperative visualization of all tumor-positive lymph nodes and distant metastases, or the decision to abort surgery in a noncurative procedure. This study implies that molecular imaging using tumor-specific targets, such as αvβ6 and CEACAM5, can help to guide this process. The main advantage of the present study is the use of tissue sections that represent a cross section of the entire pancreas which is preferred above tissue microarray (TMA) cores, as we could assess exact staining patterns more thoroughly in a larger surface and can recognize heterogeneity more reliably. This change in technique potentially explains why substantially higher expression rates are found in normal ductal pancreatic tissue compared to the literature [18, 32, 40]. To assess clinical relevance of the technique and represent the clinical situation during imaging and surgery, PDAC, lymph nodes, CP and peritumoral inflammation as well as unremarkable pancreatic parenchyma were included from the same unique patients. To our knowledge, this is the first report comparing the expression of biomarkers not only in different tissue types but also in benign and malignant lesions within the same organ from a single patient leading to the identification of ideal biomarkers for preoperative and intraoperative imaging [27, 28, 30, 41]. The results of CEACAM5 and αvβ6 in PDAC tissues are in line with most previous reports [21, 27, 30, 31]. However in contrast, Allum et al. and Jewkes et al. both reported CEA staining in CP [28, 42]. For their study, they used the monoclonal anti-CEA antibodies, 11-285-14 and 11-359-6, but it is unknown if their antibodies were directed against CEACAM5 or another subtype of CEA.

The fact that CEACAM5 could be a potential biomarker for the tumor-specific identification of PDAC would lead to other advantages. CEA is known to be present in soluble form in the serum for multiple cancer types. Recent studies have reported a positive relation between CEA levels in cancer tissue and serum CEA levels [43, 44], while other studies did not show this relation [45–48]. For now, this phenomena is mainly studied in colorectal cancer and the correlation between serum CEA and tissue CEA in PDAC is unclear, but is subject of investigation in our group.

As shown in this study as well as in literature, no individual biomarker is perfect. For most tumor types, one unique biomarker is not sufficient for exact tumor-specific identification, and therefore the potential to perform multiplexing is crucial. In this study, we showed the advantage of using probes with different fluorescent labels for multiplexing purposes, An example of a technique which is optimal for multiplexing is Raman imaging. Several different nanoparticles can be produced by modifying the Raman surface and therefore generating unique spectral signatures [49]. Unfortunately, this technique is still far from wide clinical use. To target multiple biomarkers with fluorescence imaging, diabodies are the ligand of choice now. However, if a diabody was used with one fluorescent label to target CEACAM5 and αvβ6, exact differentiation would not have been possible between normal pancreatic tissue, CP and inflammation, and PDAC since both targets have their own strengths in this differentiation. Targeting both biomarkers with different fluorescently-labeled ligands might thus be beneficial for imaging purposes.

Since the successful introduction of novel neoadjuvant regimens for PDAC, like FOLFIRINOX, resectability rates have increased up to 51% [50]. However, a major drawback of this success is that after neoadjuvant treatment conventional imaging modalities are not able to differentiate between vital tumor cells and radiochemotherapy-induced tumor necrosis and fibrosis, and are therefore no longer able to predict resectability [10]. A potential role for targeted imaging, would require that parts of the tumor that have remained vital tumor after neoadjuvant therapy would have to retained their expression levels. Avβ6 was shown to possess that capacity. CEACAM5 expression, on the other hand, was reduced compared to PDAC tissue without neoadjuvant treatment. Two potential explanations for this effect could be: (i) there is tumor heterogeneity where the subtype with CEACAM5 expression is selectively killed by the (radio)chemotherapy [51–53], or (ii) the neoadjuvant therapy has a selective effect on the cell genome [54]. However, before final conclusions can be drawn in neoadjuvant treated tissues, these results need to be validated in a larger series of patients. Another limitation of our approach in this series is that the biomarker expression before (radio)chemotherapy is unknown. However, based on the available literature and our presented results, we feel confident that biomarker expression of these tumors prior to therapy is comparable to expression in tumors that are not treated with neoadjuvant therapy.

A potential hurdle for the clinical translation of tumor-targeted imaging could be the need for agents directed against different targets in the various cancer types, which is costly and a regulatory burden. For example, the role of c-MET as a biomarker in fluorescence imaging seems to be cancer type-specific. Burggraaf et al. showed the potential of c-MET as biomarker in colon cancer [55], whereas in PDAC, its role is less clear. In this study, as with others, c-MET shows relevant overexpression in normal pancreatic tissue, excluding it as target for tumor-specific molecular imaging applications [56]. Also biomarkers targeting stromal components and microenvironment such as uPAR and Thy1 are suggested for molecular imaging of cancer [19, 57]. However, as shown, biomarkers targeting the microenvironment of PDAC, are less useful when extravasation occurs because abundant stroma exists not only in PDAC, but also in normal pancreatic tissue and especially in chronic and acute pancreatitis. Another main disadvantage of uPAR was the expression in negative lymph nodes, potentially due to its natural expression on macrophages. This is in line with a recently published review paper from Petrushnko et al [58]. Since lymph node identification was one of our main criteria, uPAR was not included in the second cohort for evaluation although it performed similar to CEACAM5 on several other characteristics.

In conclusion, both CEACAM5 and αvβ6 are promising candidates for tumor-specific molecular imaging for PDAC. Especially when used in combination, these biomarkers can discriminate between normal pancreatic tissue, CP and peritumoral inflammation, and PDAC. And in addition, can identify lymph node metastases. Also, αvβ6 can help identify vital tumor after neoadjuvant radiochemotherapy. Therefore, agents recognizing both biomarkers, combined with a different label or two separate agents, would have the potential to create a huge impact in PDAC diagnostics, therapy and patient outcome.

## MATERIALS AND METHODS

### Patient and tissue selection

Medical records and tissue specimens were retrospectively reviewed from patients who underwent pancreatic resection at the Leiden University Medical Center (LUMC) and Erasmus Medical Center (EMC) in Rotterdam between January 2011 and September 2015. Patients were selected with the histological diagnosis PDAC (n=8) or CP (n=7). In addition, normal pancreatic tissue lying adjacent to a PDAC was obtained from nine patients (n=9). Locoregional lymph nodes (n=32; n=17 tumor-negative, n=15 tumor-positive) were included from the PDAC patients to identify biomarker expression in lymph node metastasis. For our second cohort, all tissues (n=55; normal pancreatic parenchyma, inflamed pancreatic tissue, and PDAC tissue) were obtained from individual patients (n=12). All tissue samples were reviewed by a specialized pathologist before inclusion in the study. Tumor differentiation grades according to guidelines established by the World Health Organization were included. Colonic (n=3) and duodenal tissues (n=3) (from Department of Pathology, LUMC) were included as controls to assess biomarker expression in the peripancreatic organs. Patterns of expression of tumor biomarkers on pancreatic surgical specimens following neoadjuvant therapy were assessed in a small cohort (n=6) of patients who received radiochemotherapy; three cycles of gemcitabine and 15 fractions of 36 Gy radiotherapy during two cycles (PREOPANC trial).

### Antibodies and reagents

The antibodies used for immunohistochemical (IHC) staining are shown in Supplementary Table 2.

### Immunohistochemistry on tissue sections

Formalin-fixed, paraffin-embedded (FFPE) tissue blocks were collected from the Pathology Department of the LUMC and EMC. Tissue sections of 5 μm thickness were obtained from two different types of tissue blocks, one type with standard measurement of 40 × 26 mm, and the other type of 75 × 50 mm. The second type was used to determine the expression pattern across the entire specimen. All tissue slides used in the study were immunohistochemically assessed. For the second cohort of slides, additional immunofluorescence was performed. For preparation of IHC staining, the slides were deparaffinized with xylene and rehydrated in serially diluted ethanol solutions (100%-50%), followed by demineralized water according to standard protocols. Endogenous peroxidase activity was blocked by incubation in 0.3% hydrogen peroxidase in phosphate buffered saline (PBS) for 20 min. Antigen retrieval for CEACAM5, Thy1, and uPAR was performed by heat induction at 95°C using PT Link (Dako) with low-pH Envision FLEX target retrieval solution (pH 6.0, citrate buffer, Dako). For c-MET and Cath E, the high-pH Envision FLEX target retrieval solution was used (pH 9.0, citrate buffer, Dako). For αvβ6 and EGFR staining, antigen retrieval was performed with 0.4% pepsin incubation (Dako) at 37°C for 10 min.

Following antigen retrieval, the tissue sections were incubated overnight with the primary antibodies in 100 μl for standard tissue sections and 500 μl for the large tissue sections at room temperature. A pre-determined optimal dilution was used for all antibodies; anti-αvβ6 antibodies 1:800, anti-CEACAM5 antibodies 1:1000, anti-EGFR antibodies 1:100, anti-CD90/Thy1 antibodies 1:800, anti-MET antibodies 1:8000, anti-Cath E antibodies 1:1000, and anti-uPAR antibodies 1:800. The slides were washed with PBS, followed by incubation with secondary antibody for 30 minutes at room temperature. After additional washing, the staining was visualized with 3,3-diaminobenzidine tetrahydrochloride solution (DAKO, Glustrup, Denmark) at room temperature for 5 minutes and counterstained with hematoxylin for 20 seconds. Finally, the tissue sections were dehydrated and mounted in Pertex (Histolab, Rockville, MD, USA).

### IHC analysis

All stained sections with standard measurements (40 × 26 mm) were scanned and viewed at 40x magnification using the Philips Ultra-Fast Scanner 1.6 RA (Philips, Eindhoven, Netherlands). The large tissue sections were viewed under the microscope. Evaluation of the immunohistochemical staining was performed blinded independently by two observers (W.S.F.J.T. and A.F.S.). The following scoring method was used; percentage specific staining (normal/PDAC/CP) was scored as percentage of total tissue 0=<10%, 1=10-25%, 2=26-50%, 3=51-75%, 4=>75%. Staining intensity was scored as 0=none, 1=weak, 2=moderate, 3=strong (Supplementary Figure 6). Undesired staining of structures other than investigated tissues (i.e., normal/tumor/inflammation/stroma) was scored as 0=none, 1=moderate, 2=strong. In tumor tissue, a comparison was made between staining at invasive border compared to core of the tumor. This was scored as tumor core vs invasive border: 0=lower, 1=similar, 2=stronger.

### Interim analysis to assess biomarker suitability

Primary analysis of all biomarker characteristics included expression pattern, expression in normal pancreatic parenchyma, pancreatitis, PDAC and expression in tumor-positive and tumor-negative lymph nodes. Next an interim analysis of the different biomarkers was performed to assess the suitability of these markers as targets for tumor-specific molecular imaging of PDAC. After this analysis the most suitable biomarkers (αvβ6 and CEACAM5) were chosen for further assessment to specifically identify their potential in clinically relevant situations, tailored to tumor-specific molecular imaging.”

### Immunofluorescence

Double immunofluorescent staining of αvβ6 and CEACAM5 was performed on FFPE tissue sections of normal pancreatic tissue, PDAC, and CP. The slides were treated similar as described above. Antigen retrieval for both targets was performed by heat induction at 95°C using PT Link with a low-pH Envision FLEX target retrieval solution. Sections were incubated with 5% Fetal Bovine Serum in PBS for 10 minutes at room temperature prior to incubation with primary antibodies. Both primary antibodies against αvβ6 and CEACAM5 were applied overnight at room temperature. These were detected using secondary antibodies mentioned before; anti-IgG1-AF488 for αvβ6 and anti-IgG2a-AF647 for CEACAM5. After washing three times in PBS, nuclei were stained with 4′,6-diamidino-2-phenylindole (DAPI, Prolong Gold, Life Technologies) and stored at 4°C. The slides were examined using a Leica DM5500B digital fluorescence microscope (Leica Microsystems B.V., Son, the Netherlands) equipped with a Leica DFC365FX camera using LAS X software for image acquisition and processing.

### Statistical analysis

The statistical analysis was performed using SPSS version 20.0 software for Windows (SPSS, IBM Corporation, Somer NY, USA) and GraphPad Prism 6 (GraphPad, Software, Inc, La Jolla CA, USA). Mean percentage staining and difference in staining between tissues was calculated with One-way ANOVA with post-hoc Bonferroni correction. In all tests, results were considered statistically significant at the level of P<0.05.

## SUPPLEMENTARY MATERIALS FIGURES AND TABLES



## Abbreviations

PDAC: pancreatic ductal adenocarcinoma
CP: chronic pancreatitis
CEACAM5: carcinoembryonic antigen-related cell adhesion molecule 5
Cath E: Cathepsin E
EGFR: epidermal growth factor receptor
c-MET: hepatocyte growth factor receptor
Thy1: thymocyte differentiation antigen 1
uPAR: urokinase-type plasminogen activator receptor
IHC: Immunohistochemistry
