# Supplementary material for: Selection of optimal molecular targets for tumor-specific imaging in pancreatic ductal adenocarcinoma
Source: Oncotarget. 2017 May 26;8(34):56816–28. doi: 10.18632/oncotarget.18232 (PMC5593604; doi:10.18632/oncotarget.18232)
Supplement: Supplementary file 1 [file oncotarget-08-56816-s001.pdf]

Selection of optimal molecular targets for tumor-specific imaging in pancreatic ductal adenocarcinoma

SUPPLEMENTARY INFORMATION

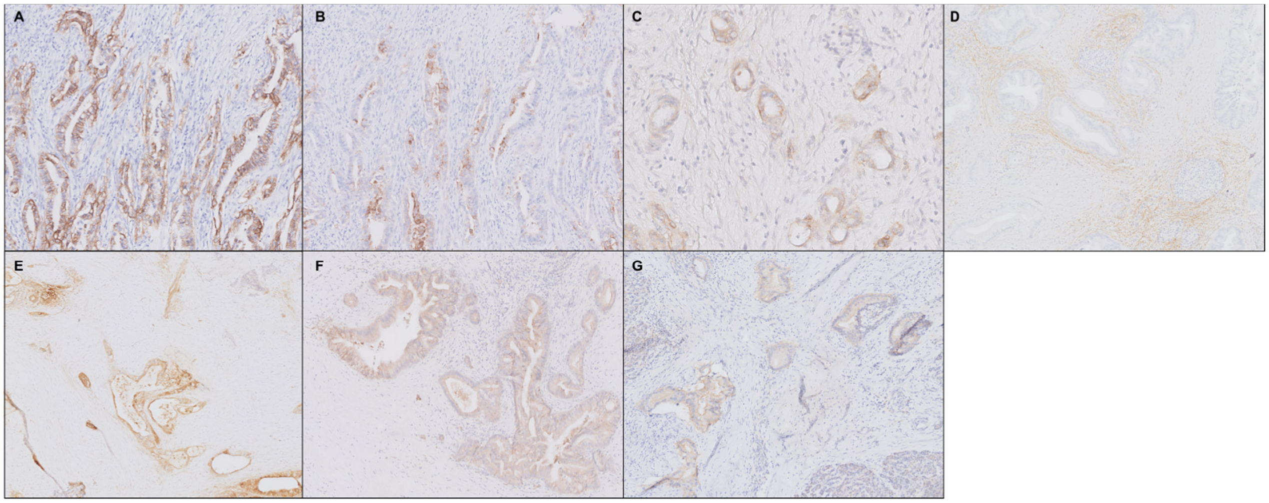

Supplementary Figure 1: Tumor heterogeneity. Representative image of PDAC staining patterns of αvβ6 **(A)** and CEACAM5 **(B)**, showing a more heterogeneous staining pattern in CEACAM5 compared to αvβ6. (10x objective).

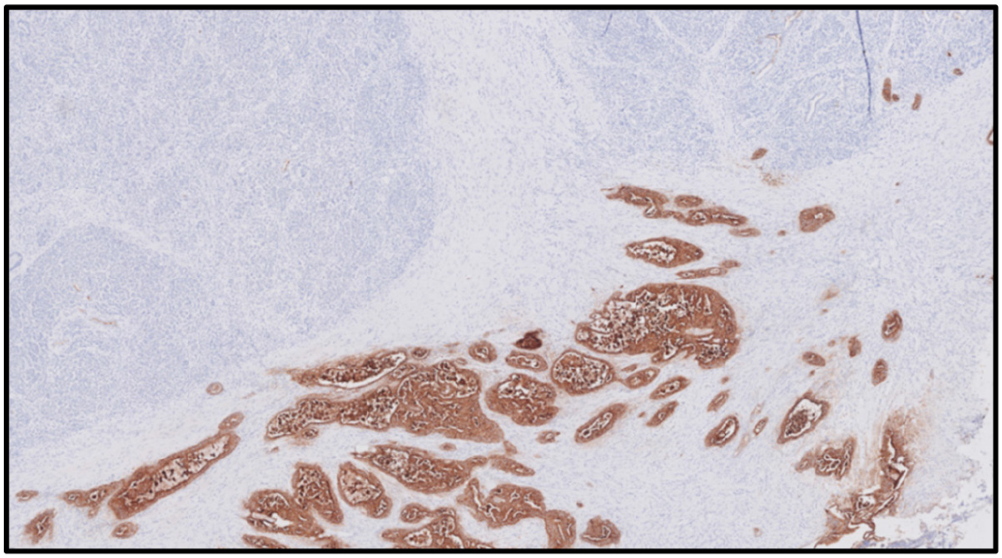

Supplementary Figure 2: Characteristic staining pattern CEACAM5. Absent CEACAM5 staining in normal pancreatic tissue even when adjacent to PDAC tissue (5x objective).

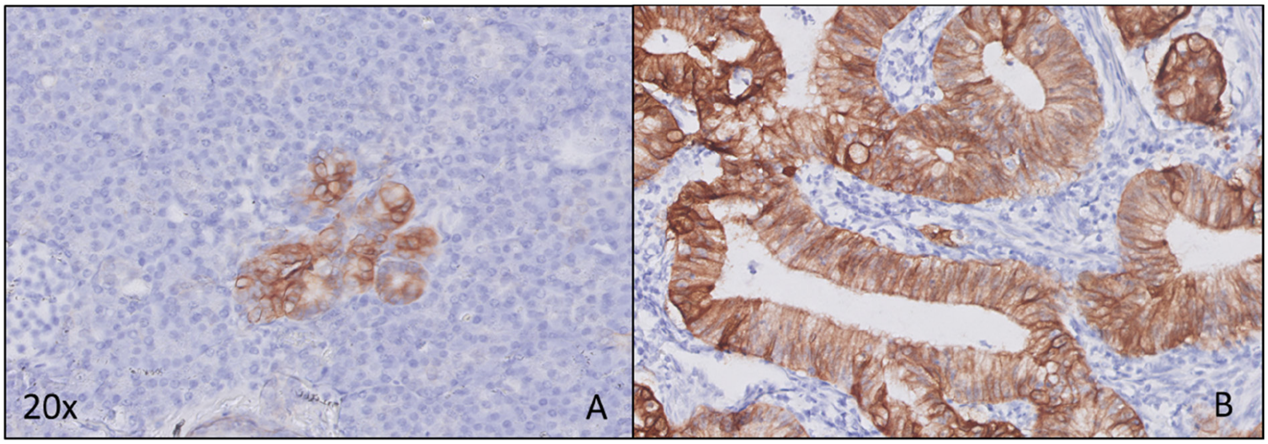

Supplementary Figure 3: Ductal staining pattern in normal pancreatic tissue versus PDAC. Representative image showing the difference in ductal αvβ6 staining in normal tissue **(A)** and PDAC **(B)**, (20x objective).

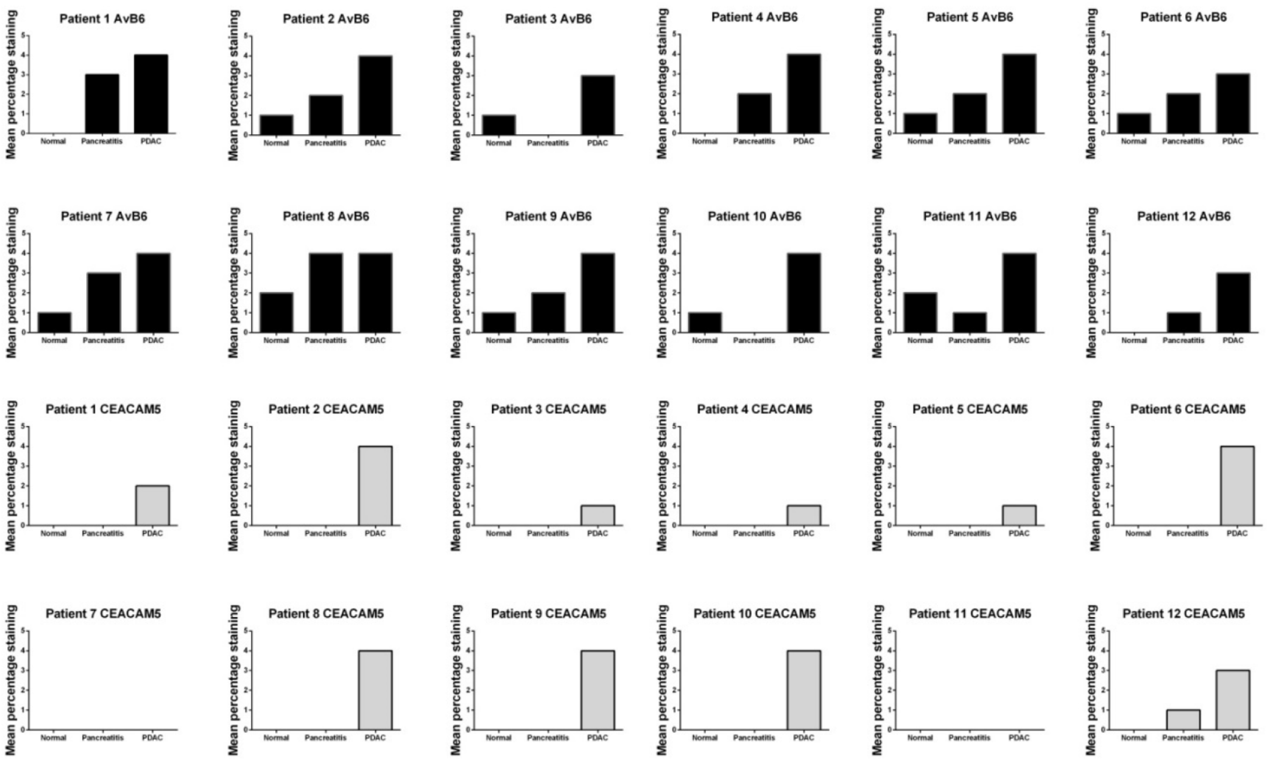

Supplementary Figure 4: Expression in validation cohort. Expression of αvβ6 and CEACAM5 within one patient in our validation cohort (n=12). The bars represent from left to right staining in normal pancreatic tissue, pancreatitis and PDAC respectively visualized per patient. For CEACAM5 there was no expression in normal pancreatic tissue and pancreatitis.

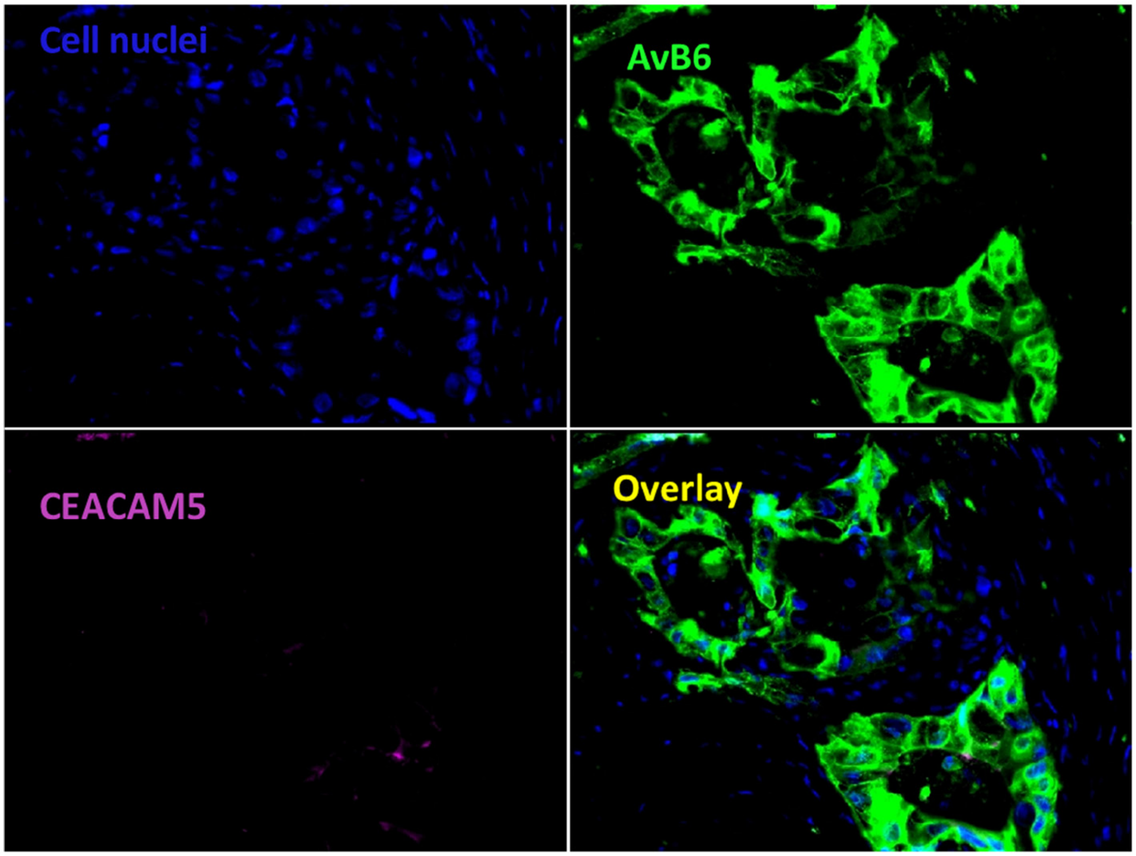

Supplementary Figure 5: Value of multiplexing. Immunofluorescent staining of αvβ6 and CEACAM5 in PDAC with a CEACAM5 negative PDAC sample. Using the double staining this tissue could be correctly identified as PDAC, however when there was only stained for CEACAM5, this spot would have been missed.

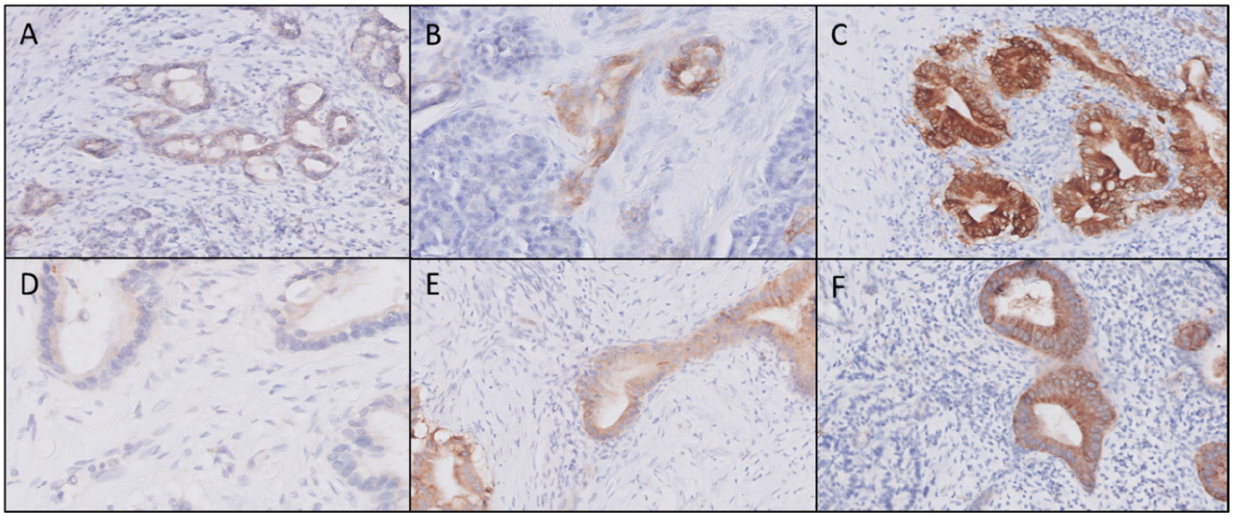

Supplementary Figure 6: Staining intensity. Representative images for each of the staining categories, divided by cell membrane **(A-C)** or cytoplasm staining **(D-F)** depending on the target of interest. Staining intensity was scored as 0=none, 1=weak **(A and D)**, 2=moderate **(B and E)**, 3=strong **(C and F)**. (Objective 20x).

Supplementary Table 1: Patient characteristics of the patients with pancreatic adenocarcinoma, and/or pancreatitis included in this study

Patient	Gender	Age	Tissue type	Histologic grade PDAC
First cohort				
**1**	M	43.0	Pancreatitis	-
**2**	M	64.8	Pancreatitis	-
**3**	M	55.7	Pancreatitis	-
**4**	V	53.4	Pancreatitis	-
**5**	M	60.4	Pancreatitis	-
**6**	V	52.9	Pancreatitis	-
**7**	V	58.3	Pancreatitis	-
**8**	V	76.1	PDAC	Moderately differentiated
**9**	M	61.7	PDAC	Moderately differentiated
**10**	M	71.9	PDAC	Poorly differentiated
**11**	V	42.9	PDAC	Well differentiated
**12**	M	67.4	PDAC	Poorly differentiated
**13**	M	72.2	PDAC	Moderately differentiated
**14**	M	58.0	PDAC	Poorly differentiated
**15**	V	75.4	PDAC	Unknown
**16**	M	76.4	Normal pancreatic tissue	-
**17**	M	71.9	Normal pancreatic tissue	-
**18**	M	72.2	Normal pancreatic tissue	-
**19**	M	43.2	Normal pancreatic tissue	-
**20**	M	43.0	Normal pancreatic tissue	-
**21**	M	70.0	Normal pancreatic tissue	-
**22**	M	70.0	Normal pancreatic tissue	-
**23**	V	79.0	Normal pancreatic tissue	-
**24**	V	57.0	Normal pancreatic tissue	-
Second cohort				
**25**	V	55.8	PDAC, inflammation, normal pancreatic tissue	Moderately differentiated
**26**	M	66.4	PDAC, inflammation, normal pancreatic tissue	Unknown
**27**	M	78.3	PDAC, inflammation, normal pancreatic tissue	Unknown
**28**	M	39.4	PDAC, inflammation, normal pancreatic tissue	Moderately differentiated
**29**	M	71.5	PDAC, inflammation, normal pancreatic tissue	Moderately differentiated
**30**	V	62.9	PDAC, inflammation, normal pancreatic tissue	Moderately differentiated
**31**	V	68.3	PDAC, inflammation, normal pancreatic tissue	Poorly differentiated
**32**	M	62.3	PDAC, inflammation, normal pancreatic tissue	Moderately differentiated
**33**	M	58.7	PDAC, inflammation, normal pancreatic tissue	Poorly differentiated
**34**	M	53.9	PDAC, inflammation, normal pancreatic tissue	Well differentiated
**35**	M	61.3	PDAC, inflammation, normal pancreatic tissue	Unknown
**36**	V	64.9	PDAC, inflammation, normal pancreatic tissue	Well differentiated

Supplementary Table 2: Antibodies and reagents used for immunohistochemistry and immunofluorescence

Antibody	IHC / IF	Catalog number	Species	Monoclonal/ polyclonal	Concentration	Dilution	Vendor
Primary antibodies							
**anti-αvβ6**	IHC / IF	6.2A1	Mouse	Monoclonal	0.5 mg/ml	1:800	Biogen Idec MA Inc., Cambridge, MA, USA
**anti-CEACAM5**	IHC	sc-23928/ CI-P83-1	Mouse	Monoclonal	0.2 mg/ml	1:1000	Santa Cruz, CA, USA
**anti-EGFR**	IHC	E30	Mouse	Monoclonal	0.34 mg/ml	1:100	Dako, Glostrup, Denmark
**anti-CD90/ Thy1**	IHC	ab92574/ EPR3132	Rabbit	Monoclonal	0.07 mg/ml	1:800	Abcam, Cambridge, UK
**anti-c-MET**	IHC	ab51067/ EP1454Y	Rabbit	Monoclonal	0.39 mg/ml	1:8000	Abcam, Cambridge, UK
**anti-Cath E**	IHC	sc-6508 / C-20	Goat	Polyclonal	0.2 mg/ml	1:1000	Santa Cruz, CA, USA
**anti-uPAR**	IHC	ATN-617	Mouse	Monoclonal	0.48 mg/ml	1:800	Kindly provided by Prof. A.P. Mazar, Northwestern University, Evanston, IL
**anti-CEACAM5**	IF	sc-59873/ 26/5/1	Mouse	Monoclonal	0.2 mg/ml	1:1200	Santa Cruz, CA, USA
Secondary antibodies							
**HRP-labeled anti-mouse**	IHC	K4001			-	Undiluted	Dako, Glostrup, Denmark
**HRP-labeled anti-rabbit**	IHC	K4003			-	Undiluted	Dako, Glostrup, Denmark
**Anti-goat**	IHC	P0449			0.55 g/L	1:100	Dako, Glostrup, Denmark
**Anti-mouse**	IF	IgG1-AF488 / A121121	Goat		2 mg/ml	1:200	Life Technologies, Thermo Fisher Scientific, Waltham, MA, USA
**Anti-mouse**	IF	IgG2a-AF647 / A21241	Goat		2 mg/ml	1:200	Life Technologies, Thermo Fisher Scientific, Waltham, MA, USA
